# Perceptions on the Effectiveness of Treatment and the Timeline of Buruli Ulcer Influence Pre-Hospital Delay Reported by Healthy Individuals

**DOI:** 10.1371/journal.pntd.0002014

**Published:** 2013-01-17

**Authors:** Marike Alferink, Tjip S. van der Werf, Ghislain E. Sopoh, Didier C. Agossadou, Yves T. Barogui, Frederic Assouto, Chantal Agossadou, Roy E. Stewart, Ymkje Stienstra, Adelita V. Ranchor

**Affiliations:** 1 University of Groningen, University Medical Center Groningen, Department of Health Sciences, Groningen, The Netherlands; 2 University of Groningen, Graduate School of Medical Sciences, Graduate School for Health Research SHARE, Groningen, The Netherlands; 3 University of Groningen, University Medical Center Groningen, Department of Internal Medicine, Infectious Diseases Service, Groningen, The Netherlands; 4 Programme de Lutte Contre la Lèpre et l'Ulcère de Buruli, Ministries of Health, Cotonou, Bénin; Fondation Raoul Follereau, France

## Abstract

**Background:**

Delay in seeking treatment at the hospital is a major challenge in current Buruli ulcer control; it is associated with severe sequelae and functional limitations. Choosing alternative treatment and psychological, social and practical factors appear to influence delay. Objectives were to determine potential predictors for pre-hospital delay with Leventhal's commonsense model of illness representations, and to explore whether the type of available dominant treatment modality influenced individuals' perceptions about BU, and therefore, influenced pre-hospital delay.

**Methodology:**

130 healthy individuals aged >18 years, living in BU-endemic areas in Benin without any history of BU were included in this cross-sectional study. Sixty four participants from areas where surgery was the dominant treatment and sixty six participants from areas where antibiotic treatment was the dominant treatment modality were recruited. Using a semi-structured interview we measured illness perceptions (IPQ-R), knowledge about BU, background variables and estimated pre-hospital delay.

**Principal Findings:**

The individual characteristics ‘effectiveness of treatment’ and ‘timeline acute-chronic’ showed the strongest association with pre-hospital delay. No differences were found between regions where surgery was the dominant treatment and regions where antibiotics were the dominant treatment modality.

**Conclusions:**

Individual characteristics, not anticipated treatment modality appeared predictors of pre-hospital delay.

## Author Summary

Delay in seeking treatment for Buruli ulcer (BU) is a major challenge in current BU control. Research to date shows that several factors relate to delay, including a lack of knowledge about BU and its treatment, beliefs in a supernatural cause of the disease, feelings of fear and worry regarding the treatment, fear of surgery, direct and indirect costs, social isolation as a consequence of unbearable costs to the patients' family, a lack of confidence in the treatment, and stigma. This study focused upon the relationship between Illness perceptions and pre-hospital delay by using the Illness Perceptions Model of Moss-Morris *et al* in a sample of healthy community members living in 3 endemic areas for Buruli ulcer in Benin. We found that a chronic timeline perspective on Buruli ulcer and a higher perceived effectiveness of the treatment were independently associated with pre-hospital delay. The available dominant treatment modality in endemic areas (surgery or antibiotics) did not influence pre-hospital delay, a finding contrary to the previous suggestion that a fear of surgery would be related to delay in presenting to the hospital. This study has identified several individual characteristics which can form the basis of future interventions.

## Introduction

Buruli ulcer (BU) is the third most important mycobacterial disease in humans today, with one of the highest prevalence rates reported in southern Benin (21.5/100,000 per year), although these numbers are probably underestimated due to the focal distribution of the disease [1]. The mode of transmission is largely unknown although skin injuries and insect bites have been suggested to play a role. Human to human transmission is considered negligible. The most important risk factor is living in tropical climates and wading in rivers or streams [2], [3]. The majority of patients in West-Africa are children [4], [5]. Buruli ulcer often starts as a firm, non-tender nodule, as plaques, or as edema. The skin breaks down into a painless ulcer with undermined edges, with the risk of complications such as osteomyelitis. After a varying period of time, a granulomatous healing response occurs, resulting in fibrosis, scarring, calcification, and contractures with residual disabilities [6]. Antimicrobial treatment (intramuscular streptomycin and oral rifampicin) has been standard since 2004; it is highly effective in the early stages of the disease, has low recurrence rates, and access to it is free of charge or at minimal costs [7]. Today, surgery is only considered for those who do not respond to antibiotics or to patients with extensive lesions. Surgery is especially important for the treatment of contractures and large skin defects where reconstructive surgery is needed [8]. Other treatment options perceived as conventional by people in Benin are self-medication and traditional (or herbal) treatment [9]–[11]. A recent study conducted in Ghana emphasizes the preference for herbal treatment, especially of patients with Buruli ulcer in an early pre-ulcerative stage [12]. Some of the major treatment centers located in the endemic south of Benin adhere to the WHO guidelines on antibiotics while others use surgery as the dominant treatment modality.

Irrespective of the treatment modalities (antimicrobial or surgical) offered, early presentation at a hospital or health care center is advantageous, because this minimizes trauma and pain, shortens admission to a treatment center, lowers the costs involved, and reduces the risk of amputation and functional limitations [6], [7], [10], [11], [13]. Despite these advantages, and the fact that people recognize BU and perceive the disease as threatening [10], [11], delay in visiting the health care center is the major challenge for national programs to fight BU. Pre-hospital delay is the time from onset of symptoms to arrival at the hospital to receive the recommended treatment [14]. Although literature is limited, previous explorative studies indicate several internal and external factors related to delay, including a lack of knowledge about BU and its treatment, beliefs in a supernatural cause of the disease, feelings of fear and worry regarding the treatment, fear of surgery, direct and indirect costs, social isolation as a consequence of unbearable costs to the patients' family, a lack of confidence in the treatment, and stigma [6], [10], [15], [16].

National BU control programs from several endemic countries initiated awareness raising campaigns followed by active case-finding strategies, which have shown to be effective in Benin [17], Côte d'Ivoire [18], Ghana [19], [20] and the Democratic Republic of the Congo [21]. Such programs seem to diminish delay, however, the question remains why some patients do while others do not delay in presenting to the hospital.

Based on previous psycho-social research on delay in Buruli ulcer,we hypothesize that despite economic, social factors and a lack of knowledge about BU and its treatment, cognitive and emotional factors play a role in delay, e.g. beliefs in a supernatural cause of the disease, feelings of fear and worry regarding the treatment, fear of surgery and a lack of confidence in the treatment. The studies that addressed the psycho-social factors in Buruli ulcer thus far, did not go into detail about the cognitive and emotional representations people have of Buruli ulcer.

Leventhal's commonsense model of illness (CSM) is a self-regulation model which describes how people respond to a health threat or illness. One assumption of the CSM is that people form cognitive and emotional representations to the illness. People will try to manage these emotions and cognitions by coping efforts. These coping efforts lead the person to take action e.g. visiting a doctor, taking medication. The coping strategy used, depends on their representation of the illness. This model is advantageous over other models such as the Access framework of health care utilization [22] and Anderson's model of health care utilization [23] in that it is unique to the individual and disease-specific.

**Figure 1:**
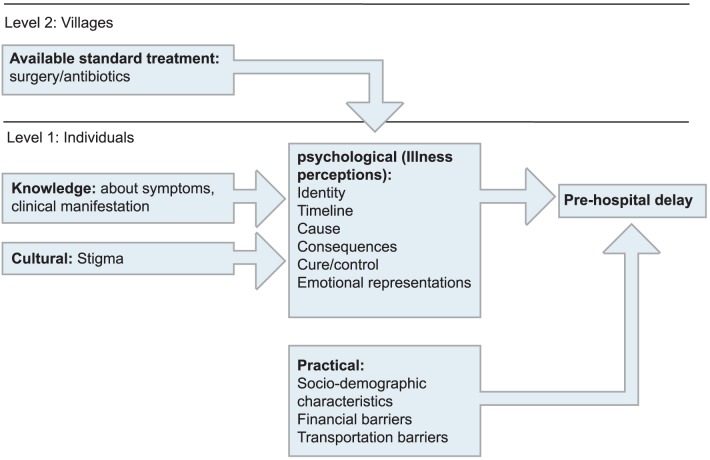
Model with level 1 and 2 factors potentially related to pre-hospital delay.

The model describes a process in which the cognitive and emotional responses to an illness occur in parallel. The cognitive part is made up of perceptions on the identity, timeline (acute/chronic or cyclical), causes, consequences, coherence, control (treatment control/personal control) of the disease, the emotional part comprises emotional representations (figure 1). Perceptions on the identity of the disease contain ideas on symptoms attached to BU, for example, induration of the skin and underlying tissue. Timeline perceptions are ideas on the acuteness, chronicity or cyclical timeframe of the illness. Causal beliefs comprises views on the factors that caused Buruli ulcer, which are divided into biological, emotional, environmental and psychological causes. Consequences refer to the impact BU has on daily life, social and occupational functioning. The extent to which people feel they understand their disease is measured by the coherence subscale, and the control subscale is the extent to which people think they can influence the course of their disease and perceive treatment to be effective.

Basic knowledge about the disease and cultural factors (stigma) are suggested to influence illness perceptions and therefore, influence pre-hospital delay indirectly, while practical factors (time and distance to the hospital) are known to directly influence pre-hospital delay. We expect the regionally determined differences in dominant treatment modality to influence individuals' perceptions about BU, and therefore, influence pre-hospital delay indirectly (figure 1).

Illness perceptions have shown to be related to delay for different chronic illnesses [24], as well as infectious diseases such as tuberculosis [25] and they appear to exist in healthy individuals as well [26]. Illness perceptions are especially relevant in Buruli ulcer, because literature describes BU as being associated with cognitions such as worries about (economic) consequences of the treatment, ideas on causal mechanisms, major social (stigma) as well as general consequences on ones lives. Therefore, it seems important to ask questions about how an individual thinks about Buruli ulcer, how they would cope and the sense they make of it. Illness perceptions can quantitatively be measured by the widely used and validated Illness Perceptions Questionnaire [24], which can be adapted to a specific illness.

Our target group is healthy community members living in high risk areas for BU, in order to capture the perceptions and the future pre-hospital delay of those who are at risk for contracting the disease. The reason for choosing this group instead of actual cases, is that we are interested into the beliefs of potential patients irrespective of their treatment choice. Since actual cases already made their decision, namely; going to the hospital, we would only have captured the beliefs of this group while we are especially interested in the beliefs of those who would not present to the hospital, because this group is at risk for pre-hospital delay.

The first aim was to explore to what extent individual characteristics were related to future pre-hospital delay. The second aim was to explore whether the type of available dominant treatment modality influenced individuals' perceptions about BU, and therefore, influenced pre-hospital delay (figure 1).

## Methods

### Participants and procedure

#### Sample and sampling

Data for this cross-sectional study were collected between September and November 2010. A total number of 130 respondents, aged ≥18 years, currently not diseased with or treated for any kind of disease, and without any history of BU were included. All participants resided in a rural area.

**Figure 2:**
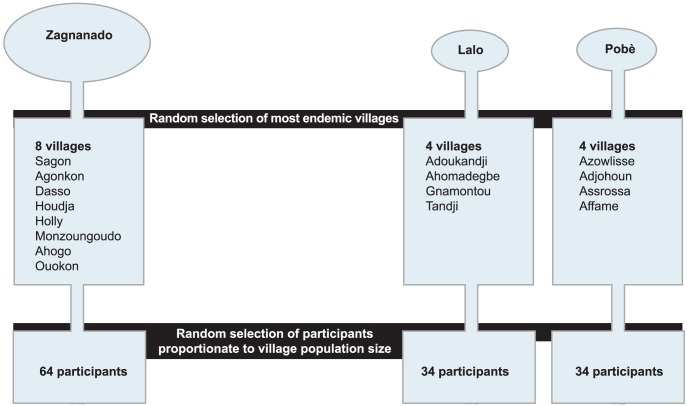
A multistage sampling procedure.

Participants from a region where surgery was the dominant treatment (group 1) and from two regions where antibiotic treatment was the dominant treatment (group 2) were included. The rationale for choosing 3 regions (2 antibiotic-modality and 1 surgery-modality), was that this provided the opportunity to also look at two regions with the same treatment modality. If the differences between the antibiotic-modality and surgery-modality would have been large, and the differences between the two antibiotic-modality regions would have also been large, it is more likely that differences are due to other factors. However, if latter differences were small, it is more likely that differences are due to treatment modality differences. Group 1 included 64 participants living in villages around Zagnanado, group 2 included 34 participants living in villages around Lalo and 32 participants living in villages around Pobè. Figure 2 presents the multi-stage sampling procedure [27] to select participants. Within each of the three regions, the most endemic villages were selected. In each endemic village, 4 or 5 neighborhoods were randomly selected to maximize the spatial distribution of the participants' houses. Five (villages of Tandji, Ahomadegbe) to 16 (villaged of Adoukandji) participants were approached for participation, the number depending on the population size of the village.

#### Interviewers

A trained male and a female interviewer performed the selection procedure and the data collection by a semi-structured interview, and were assigned to interview approximately equal numbers of men and women. Both were university educated (sociology and psychology), able to speak Fon, familiar with the regions under study and experienced with performing interviews. They engaged in a two-day interview training program covering the study procedures, general interview skills, and possible biases in interviewing. To ensure reliability between the interviewers, we performed a pilot study (data not presented) in which discrepancies on the content of the questions, the translation, the interpretation of the answers, and the non-verbal behavior were discussed among the interviewers, translators and researchers. An independent observer regularly listened while the interviewers performed their work in order to maintain full agreement on translation.

#### Procedure

After arriving at a selected endemic village, the chief of the village was asked for permission to perform interviews. The two interviewers started at the center of the village and chose a walking direction randomly by spinning a pen. Once the direction was chosen, the first interviewer selected the first house in the first selected neighborhood that appeared. The second interviewer went to the next selected neighborhood, choosing the first house encountered. Arriving at a house, the interviewer approached the first adult met. He/she asked questions designed to ascertain whether the person met the inclusion criteria (described under “*Sample*”). The interview began with a verbal informed consent, followed by an introduction, and a set of standard questionnaires (described under “*Measurements*”). The interview was carried out in a private setting (inside the participant's house, or a quiet place) directly after agreement to participate, had a duration of approximately 1½ hours and participants received a small compensation for the time spent. A researcher (MA) was present to monitor the selection procedure and interviews and to answer questions.

### Measurements

Questionnaires were translated and back-translated from French to Fon, the local language in this area of Benin.

#### Pre-hospital delay

**Figure 3:**
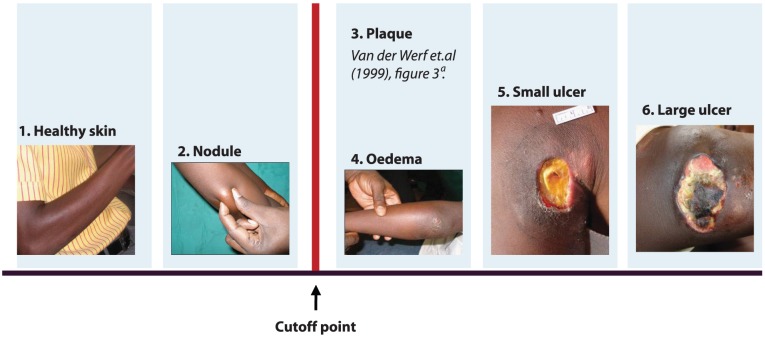
Skin pictures (WHO) shown to respondents to estimate pre-hospital delay. ^a^ From The Lancet, 354, Tjip S Van der Werf, Winette TA Van der Graaf, Jordan W Tappero, Kingsley Asiedu, Mycobacterium ulcerans infection, 1013–1018, 1999 [39].

Pre-hospital delay was measured by showing participants six consecutive pictures; a healthy skin picture, early nodule, plaque, oedema, small ulcer and a large ulcer (figure 3), all BU-pictures published by the WHO. Participants were asked to imagine that their skin looked like the skin in the pictures. After showing the picture, the question “What would you do?” was asked, followed by the five possible answers; (1) “continue with daily life as usual,” (2) self-medicate, (3) go to a traditional healer, (4) go to a hospital/health center, and (5) “do something else”. Pre-hospital delay was dichotomized, based on expert opinion (TW; YS); respondents who indicated to present at the hospital/health center if their skin would look like the skin on picture 1 or 2 were non delayers, respondents who indicated that they would go to the hospital no earlier than having an ulcer such as on picture 3 to 6 were delayers.

#### Illness perceptions

The Revised Illness Perception Questionnaire (IPQ-R) (Figure S2), described by Moss-Morris [24] was used to assess the six components of Leventhal's common sense model of illness representations. It was adapted for the population under study as follows. Since patients perceive traditional treatment, self-medication, and medical treatment as common treatment options, it was extended with six statements on the perceived effect of each of those treatments. The original list of 18 causal beliefs was extended with 8 items representing likely causes of BU as perceived by the study population, such as contaminated water, walking in the mud or an insect bite. Furthermore, the wording was adapted for healthy people. Instead of statements concerning actual experiences of the illness, statements about a person's belief should he/she have BU were provided, for example, “If I had Buruli ulcer, I would expect to have it for the rest of my life” (Item 1). Because the identity component is measured by a simple summation of self-reports of experienced symptoms and healthy individuals do not experience symptoms, this part was not used. All components of the IPQ-R were rated on a 5-point Likert scale, ranging from *strongly disagree* to *strongly agree*, with exception of one final open question concerning the three most important causes of BU.

Internal reliability and different aspects of the validity of the IPQ-R were sufficient across different patient groups [24] and in healthy individuals [26]. By performing a Principal Component Analysis (PCA) on our data, five of the theoretically derived factors labeled timeline acute/chronic, consequences, illness coherence, timeline cyclical, and emotional representations could be confirmed; loadings were sufficient and Cronbach's alpha ranging from .65 to .85 indicated a good internal reliability. The factor ‘controllability’ (items 12 to 23) could not be confirmed by our data; factor loadings were low, or items loaded on different factors. A second PCA was performed for these items separately, resulting four new subscales, respectively perceived effectiveness of treatment, personal control, influence on course and helplessness. A multigroup method (MGM) analysis [28] on items 12 to 23 confirmed this classification (manuscript in preparation).

#### Socio-demographic and economic factors

Participants were requested to provide basic demographic information as to gender, age, ethnicity, religion, urban or rural residence, and region where participants lived, income, occupation, employment status, distance to the hospital, and level of education. Means of transportation and the self-perceived time from home to hospital were assessed (data not presented). Barriers in presenting to the hospital were assessed by showing the same 6 pictures as described under subheading ‘*Pre-hospital delay*’ and asking participants to indicate what would prevent them from going to the hospital when their skin would look like the skin on each of the pictures.

#### Knowledge about Buruli ulcer

Knowledge was measured by two questions. The first question captured knowledge about transmission (‘*Is BU transmissible from person to person*), the second question addressed the direct costs of treatment (‘*Can BU be treated for free?*’). Knowledge was defined as ‘high’ when both questions were answered correctly, and low when 1 or 2 questions were answered inaccurately.

#### Ethical considerations

No ethical approval was needed for this study. Verbal consent was given by all of the individual participants after the explanation of the study aims, voluntary participation, free withdrawal at any moment, anonymous data processing, and the opportunity to ask questions.

### Statistical analysis

Analysis was performed using SPSS Statistics 17.0 and MLwiN 2.24. Means and standard deviations were calculated per region and for the total group of participants. Inter-relationships were indicated by Spearman's rho for ordinal variables and Chi-square statistics for the binary outcome; pre-hospital delay. A logistic regression analysis was performed with pre-hospital delay as outcome variable. All psychological, cultural and practical factors significantly related to pre-hospital delay, as well as knowledge on BU were entered. A second logistic regression analysis was performed with only the most important predictors for delay, starting with cognitive illness representations, followed by emotional illness representations, practical factors and knowledge on BU.

A multilevel logistic regression model with individuals (level 1) nested in villages (level 2) was used to examine the effect on pre-hospital delay. The factors shown to be related to pre-hospital delay in the logistic regression analysis were selected as individual-level characteristic. Dominant treatment modality was added as village characteristic. The final model selection was based on the estimate of the variables (*p*≈(approx.) .05) Continuous variables were centered around 1 in order to ease interpretation. Wald statistics were used to test the significance of the coefficients. High correlations between independent variables were examined on multicollinearity.

## Results

### Sample characteristics

An overview of sample characteristics and health-related factors for the total group (*n* = 130) and per region (Lalo, Zagnanado, Pobè) are presented in table 1. No significant regional differences were found on background variables.

**Table 1:** Sample characteristics.

	Total	Group 1: Antibiotics		Group 2: Surgery
		Lalo	Pobè	Zagnanado
	(*n* = 130)	(*n* = 34)	(*n* = 32)	(*n* = 64)
Female sex, No. (%)	52 (40.0)	15 (44.1)	12 (37.5)	25 (39.1)
Age, mean, (sd)	36.3 (13.2)	31.5 (11.4)	38.6 (15.0)	37.6 (12.9)
Religion, No. (%)				
Traditional	29 (22.3)	15 (44.1)	4 (12.5)	10 (15.6)
Catholic	39 (30.0)	2 (5.9)	17 (53.1)	20 (31.3)
Protestant	15 (11.5)	1 (2.9)	0	14 (21.9)
Muslim	3 (2.3)	0	2 (6.3)	1 (1.6)
Other religions	44 (33.8)	16 (47.1)	9 (28.1)	19 (29.7)
Ethnicity, No. (%)				
Fon	119 (92.2)	25 (75.8)	31 (96.9)	63 (98.4)
Adja	10 (1.6)	8 (24.2)	1 (3.1)	1 (1.6)
Level of education, No. (%)				
Never attended	69 (55.6)	20 (64.5)	12 (37.5)	37 (60.7)
Incomplete primary	29 (22.4)	5 (16.1)	11 (34.4)	13 (21.3)
Completed primary	8 (6.5)	3 (9.7)	2 (6.3)	3 (4.9)
Incomplete secondary	16 (12.9)	3 (9.7)	6 (18.8)	7 (11.5)
Completed secondary or more	2 (1.6)	0	1 (3.1)	1 (1.6)
Employment status, No. (%)	130 (100)	34 (100)	32 (100)	64 (100)
Total monthly family income (€), Mean (sd)	37.55 (32.77)	33.68 (26.82)	33.66 (17.00)	42.29 (40.57)
General health status (scale 1–5) Mean (sd)	3.4 (0.83)	3.8 (0.74)	3.4 (0.75)	3.3 (0.86)
Health insurance YES, No. (%)	6 (4.6)	3 (8.8)	1 (3.1)	2 (3.1)

### Pre-hospital delay

Forty five percent of the participants (*n* = 58) responded to the pictures in such a way that they were classified as ‘delayers’, while 55% (*n* = 72) were classified to be ‘non-delayers’. There were no regional differences (*χ^2^* degrees of freedom (*df*) = 1) = 2.65, *p* = .26) or differences between the antibiotics and the surgery group (*χ^2^* (*df* = 1) = 2.46 *p* = .12) on pre-hospital delay.

### Knowledge on BU

32% of the respondents believed inaccurately that BU was transmissible from person to person and almost half of our respondents (48.4%) believed inaccurately that there were direct costs involved in treatment. No regional or treatment differences were found on knowledge on BU (resp. *χ^2^* (*df* = 1) = 1.95 *p* = .16 and *χ^2^* (*df* = 2) = 1.95 *p* = .38).

### Relationship between potential predictors and pre-hospital delay

Table 2 presents inter-correlations between explanatory variables, and their relationship with pre-hospital delay. There was a significant association between delay and eight subscales of the illness perception – i.e., timeline acute/chronic, illness coherence, effectiveness of treatment, effectiveness of alternative treatment, personal control, emotional representations and ‘chance’ as a cause of BU. Knowledge on BU was significantly associated with delay. Effect sizes for the mean difference on delay were large for the perceived effectiveness of treatment, personal control and timeline cyclical (table 2, bottom row).

**Table 2:** Inter-correlations between explanatory variables, and pre-hospital delay.

	1.	2.	3.	4.	5.	6.	7.	8.	9.	10.	11.	12.	13.	14.	15.	16.
1. Pre-hospital delay	-															
2. Timeline cyclical	.37**	-														
3. Timeline acute - chronic	−.14	−.13	-													
4. Consequences	.01	−.05	.36**	-												
5. Illness coherence	.29**	.47**	−.33**	−.14	-											
6. Perceived effectiveness of treatment	.32**	.27**	−.20*	−.16	.41**	-										
7. Personal control	.44**	.51**	−.21*	−.06	.46**	.41**	-									
8. Influence on course	.12	.15	.04	.11	.06	−.15	.06	-								
9. Helplessness	.06	.01	.04	.02	.08	.07	.12	−.06	-							
10. Emotional representations	−.23**	−.45**	.29**	.12	−.40**	−.33**	−.41**	−.24**	−.02	-						
11. Perceived effect of alternative treatment	.08	.03	−.19*	−0.1	.13	.29**	.11	−.03	−.03	−.08	-					
12. Perceived effect of treatment at a health care center	−.05	.14	.14	.25**	−.08	−.17	.01	.16	−.06	−.04	−.44**	-				
13. Accurate causes	.14	.37**	.11	.30**	.04	.10	.11	−.09	−.12	.02	−.09	.19*	-			
14. Chance as cause	−.25	.15	.17	.13	−.34**	−.26**	−.53**	−.10	−.22*	.37**	−.07	−.08	−.09	-		
15. Behavior as cause	.00	−.55**	.15	.17	−.29**	−.19*	−.25**	−.03	−.20*	.33**	−.05	.08	.08	.38**	-	
16. Knowledge on BU	−.15	−.32**	.24**	.10	−.42**	−.20*	−.24**	−.02	−.07	.30**	−.22*	.09	−.03	.30**	.10	
Effect size^a^	-	L	M	S	M	L	L	S	S	M	M	S	S	M	S	S

** ; significant at 0.01,

* ; significant at 0.05,

a Cohen's measure of effect size for mean difference on ‘pre hospital delay’: S = small (.2), M = medium (.5), L = large (.8).

The results of the logistic regression analysis (table 3) presents the model with the best fit, *Model X^2^* (*df* = 5) = 47.64, *p*<.001; (Hosmer & Lemeshow test *X^2^* (*df* = 8) = 7.11; *p* = 5.25). The most important predictors for the outcome ‘pre-hospital delay’ were personal control and timeline acute-chronic. If personal control increased by one unit (people perceive 1 unit more control over the illness; scale range 1–5) people are 2.10 times more likely to show pre-hospital delay. If timeline increased by one unit (people perceive the illness 1 unit more chronic in timeline; scale range 1–5), the probability to be delayed increased twice (Cox & Snell = .29, Nagelkerke *R^2^* = .43.)

**Table 3:** Logistic regression analysis (enter method) on pre-hospital delay.

	B (SE)	Wald	Sig.	95% C.I. for Odds ratio		
				Lower	Odds Ratio	Upper
Timeline acute-chronic	.68 (.32)	4.38	.04	1.04	1.96	3.69
Personal control	.74 (.35)	4.59	.03	1.07	2.10	4.14
Effectiveness of treatment	.71 (.39)	3.38	.07	.95	2.04	4.38
Constant	−4.60 (1.01)	20.78	.00		.01	

### The effect of the dominant treatment on individual factors related to pre-hospital delay

Table 4 presents the multilevel model with three dimensions of the illness perceptions as level 1 variables and dominant treatment as level 2 variable. The coefficient for dominant treatment was not significant, which means there is no difference between the region where surgery is the dominant treatment modality and regions where antibiotics are the dominant treatment on pre-hospital delay.

**Table 4:** Multilevel model with level 1 and level 2 variables.

	Main effects	
*Fixed effects*	*Coefficient*	*S.E.*
Level 2		
Intercept	−2.58	
Dominant treatment	−.80	.55
Level 1		
Timeline acute - chronic	.72	.36*
Personal control	.84	.44
Effectiveness of treatment	.91	.40*
*Random effects*	*Variance*	*S.E.*
Level 2 var. 2nd order/PQL	.37	.40

* Wald statistic used to test the significance of the coefficients.

Two level 1 dimensions - the effectiveness of treatment and timeline acute-chronic, had significant coefficients. This reflects an increased probability of pre-hospital delay when the score of one of these dimensions increases, adjusted for the other level 1 and 2 variables. The adjustment means that the effect on pre-hospital delay is consistent, regardless of the other variables in the model.

We take an example of a person who lives in a region where surgery is the dominant treatment, perceives the treatment as not effective and thinks BU is acute in timeline (which is what most people believe). If this persons' score on the personal control dimension is low (e.g. *There is nothing which I can do to control my symptoms -*item 12), the probability of delay is 28%. When this persons' score on the personal control dimension is high (e.g. *I have the power to influence my illness* - item 16) the probability of delay is 92%. The proportion variance present at level 2 is 0.10 (0.374/0.374+3.29 [29]), which is twice as large as the proportion of unexplained variance in a model with no variables included (0.173/(0.173+3.29) = 0.05; data not presented). The explained level 2 variance by the full model presented in table 4 is 5%.

The multilevel model indicates that there were non-significant, small dominant-treatment modality differences (level 2) on pre-hospital delay. However, the illness perceptions dimensions: effectiveness of treatment, and timeline acute-chronic (level 1) were more important.

## Discussion

The results of this study suggest that psychological factors were predictors for pre-hospital delay, and not factors related to the dominant treatment available for BU (surgery or antibiotics). People who perceived BU as chronic in timeline, perceived treatment as effective or perceived higher personal control over the disease had a higher probability of delay. The dominant treatment available (surgery or antibiotics) in endemic regions in Benin did not show any effect on pre-hospital delay or on the individual characteristics related to pre-hospital delay.

Limited research is performed on the relationship between the type of treatment offered in a certain region and the amount of pre-hospital delay of individuals living in these regions. In a systematic review on factors related to treatment adherence in tuberculosis patients by Munro *et al* [30] reviewing studies from Asia, Africa, Europe and the USA, no influence of geographic location or type of treatment program was observed on treatment adherence. Instead, a number of structural, social, health service and personal factors correlated with treatment adherence. It is plausible that in our study, despite knowing someone with BU and living in highly endemic areas, respondents were not aware of the treatment modality provided in their region and that this was the reason that treatment modality was not related to pre-hospital delay.

In our study, illness perceptions were important for pre-hospital delay. People who believed the illness to have a chronic timeline, were more likely to delay. It is known that people who believe an illness to be chronic are more likely to attribute it to causes such as health habits, while people who believe an illness to be acute, are more likely to see a virus or bacterial agent as the cause. Our results are supported by a meta-analysis of Figureas and Alves on illness perceptions in healthy individuals [26]. They report a chronic timeline perception to account for a significant proportion of variance in attitudes towards preventive health behavior, irrespective of the experience of the illness.

Participants in our study who believed more in the effectiveness of treatment were more likely to delay, a finding which is in line with a recent study of Peeters *et.al* (2012) [31], who describe the length and complexity of patients treatment choices as result of their determined search for effective treatment. Some patients in their study experienced financial and professional loss and social isolation due to their search for effective treatment. They conclude that the overall difficulty of finding successful treatment is an important factor for late arrival at treatment centers. A similar explanation might be at stake here. An alternative explanation is that due to the high inter-correlation between the dimensions ‘Effectiveness of treatment’ and ‘effectiveness of alternative treatment’, people interpreted the word ‘treatment’ as alternative treatment and the individuals who found this effective were more likely to delay. This is in line with previous findings of Stienstra *et al* (2002), and Brienza *et al* (2002) [6], [32].

A stronger believe in the controllability of the illness by one's own behavior was related to more pre-hospital delay. A review of Brienza *et al* [32], claims that personal control is related to adaptive outcomes such as changing health behavior. An explanation for the relationship found in our study could be that individuals who perceive more personal control are more likely to take a situation into their own hands, decide to seek help in alternative treatment or engage in self-medication and therefore, delay in presenting in the hospital.

A strength of the CSM is that it is a dynamic model which is unique to the person. The model is applicable to specific illnesses, as opposed to more generalised health behaviour models such as the Access framework of health care utilization [22] and Anderson's model of health care utilization [23]. Furthermore, it has proven to predict a number of health behaviors such as adherence to treatment, treatment attendance, delay and recovery from illness. A strength of using the IPQ-R in measuring illness perceptions is that items relevant to specific illnesses can be added while maintaining psychometric validity.

Limitation of the use of the CSM in our study is that it describes illness perceptions and coping as being a dynamic process, however, when using the IPQ-R in a cross-sectional study, this process view is not taken into account. Although our study is useful in identifying factors that may impact on delay, they do not test for causal relationships. Another limitation is that knowledge about the psychometric properties of the IPQ-R in African populations is limited, since the instrument was developed and used mostly in European populations. To our knowledge, there is one study reporting on the IPQ-R in a from Africa origin diabetes population [33]. We found similar patterns of inter-correlations between subscales of the IPQ –R with this study. Timeline cyclical was positively related to consequences, illness coherence and personal control. Illness coherence correlated positively with personal control and negatively with emotional representations. Timeline acute-chronic correlated positively with consequences and emotional representations and negatively with personal control and illness coherence. The positive relationships between timeline and consequences, the negative relationship between timeline and cure/control (personal control and effectiveness of treatment) and cure/control and consequences were also similar to previous psychometric studies [34]. There were also discrepancies which could be due to differences across varying disease types, implying that outcomes are specific to specific diseases.

The relationships established in this cross-sectional study were based upon an estimated measure of pre-hospital delay of healthy people. Forty five percent of our participants expected to show up in a late stage (picture 3, 4, 5 or 6 in figure 3), while 55% expected to show up at the hospital in an early phase (picture 1 and 2 in figure 3). When considering the accuracy of this estimation, some attention should be paid to the possibility of a self-serving bias [35]. This concept assumes that people tend to overestimate their own behavior, while accurately predicting others' behavior [36]. Therefore, the proportion of people expecting to show pre-hospital delay in our study might be an underestimation of the real proportion, strengthening the relevance of factors we found to be related to pre-hospital delay.

A strength of this study is that above stated relationships were established by quantitative, individually derived data with standardized instruments, adding information to the results of previous studies using different approaches. Furthermore, the geographical distribution where participants resided, and the high (99%) response rate, contributed to the representativeness of the sample. Finally, an approach in which native-looking and native-speaking interviewers performed the interviews contributed to the quality of the data.

A limitation was the relatively small sample size (*n* = 130) which might have contributed to the lack of a statistically significant impact of the dominant treatment modality differences on pre-hospital delay, although the required sample size of 56 participants in each group should have given sufficient power to detect a meaningful difference on delay. Furthermore, the cross-sectional design of the study restricts the results to associations, and although predictors for delay are suggested, these are potential predictors for which no causal interference can be made. Another limitation is our choice for not taking previous medical history, which is often a contributing factor for delay [37] into account. Another limitation is the relatively low number of level 2 entities (17 villages). Scherbaum et al recommend a minimum of 30 level 2 units in for performing a multilevel analysis [38].

### Conclusion

Our findings add to literature the importance of individual characteristics in explaining pre-hospital delay, above and beyond practical (means of transportation and the self-perceived time from home to hospital), socio-demographic and economic factors, and knowledge on the disease. The measures (IPQ-R) in this study are new for this population, and further research is needed to explain some of the counterintuitive findings such as the relationship between personal control and pre-hospital delay.

Further research is also needed in order to explain whether the illness perceptions of healthy individuals predict delay using longitudinal designs. This cross-section study cannot explain why people report late, because this would need an approach in which a solid qualitative method is used that departs from these predictors and systematically seeks for reasons.

We suggest studying this with a multilevel design which incorporates sufficient level 2 entities and by using neighborhoods instead of villages as the aggregated level. We expect that neighborhoods reflect a more appropriate macro level, because differences in and between neighborhood with respect to beliefs on the effectiveness of treatment are more relevant than differences on a village level. Such studies may help in identifying factors to focus upon in community programs aiming at reducing pre-hospital delay.

## Supporting Information

Figure S1 **What is already known and what this paper adds.**
(DOCX)

Figure S2 **IPQ-R reworded for healthy individuals.**
(DOCX)
