# Supplementary material for: Perceptions on the Effectiveness of Treatment and the Timeline of Buruli Ulcer Influence Pre-Hospital Delay Reported by Healthy Individuals
Source: PLoS Negl Trop Dis. 2013 Jan 17;7(1):e2014. doi: 10.1371/journal.pntd.0002014 (PMC3547863; doi:10.1371/journal.pntd.0002014)
Supplement: Figure S1 — What is already known and what this paper adds. (DOCX) [file pntd.0002014.s001.docx]

**What is already known and what this paper adds.**

| 1. Delay in presenting to the hospital with Buruli ulcer is a serious problem. |
| --- |
| 1. Research to date shows that several factors relate to delay, including a lack of knowledge about BU and its treatment, beliefs in a supernatural cause of the disease, feelings of fear and worry regarding the treatment, fear of surgery, direct and indirect costs, social isolation as a consequence of unbearable costs to the patients’ family, a lack of confidence in the treatment, and stigma. |
| 1. Literature shows that illness perceptions affect delay in seeking help and current study was aimed at testing the relationship between individuals’ Illness perceptions and pre-hospital delay by using the illness perceptions model of Moss-Morris et al. |
| 1. We found that a chronic timeline perspective on Buruli ulcer and a higher perceived effectiveness of the treatment were independently associated with pre-hospital delay. |
| 1. The available dominant treatment modality in endemic areas (surgery or antibiotics) did not influence pre-hospital delay, a finding contrary to previous suggestion that a fear of surgery would be related to delay in presenting to the hospital. |
| 1. This study has identified several individual characteristics which can form the basis of future interventions. |
