# Supplementary material for: Perceptions on the Effectiveness of Treatment and the Timeline of Buruli Ulcer Influence Pre-Hospital Delay Reported by Healthy Individuals
Source: PLoS Negl Trop Dis. 2013 Jan 17;7(1):e2014. doi: 10.1371/journal.pntd.0002014 (PMC3547863; doi:10.1371/journal.pntd.0002014)
Supplement: Figure S2 — IPQ-R reworded for healthy individuals. (DOCX) [file pntd.0002014.s002.docx]

| **PARTIE 3 – VOTRE OPINION SUR L’ULCERE DE BURULI**  **Nous sommes intéressés par votre opinion personnelle ou comment vous voyez l'ulcère de Buruli. Ce qui signifie que pour tous les états, vous devez imaginer que vous avez l’UB.** |
| --- |

|  | VOTRE POINT DE VUE  S`IL VOUS ARRIVAIT DÁVOIR L ’ULCERE DE BURULI | PAS DU TOUT D’ACCORD | PAS D’ACCORD | NI D’ACCORD NI PAS D’ACCORD | D’ACCORD | TOUT A FAIT D’ACCORD |
| --- | --- | --- | --- | --- | --- | --- |
|  | ***Si j'aurais L'ulcère de Buruli ... ....*** | | | | | |
|  | Ma maladie ne va pas durer longtemps |  |  |  |  |  |
|  | Ma maladie est susceptible d’être permanente plutôt que temporaire |  |  |  |  |  |
|  | Ma maladie va durer longtemps |  |  |  |  |  |
|  | Cette maladie va rapidement passer |  |  |  |  |  |
|  | Je pense que je vais la trainer tout le restant de ma vie |  |  |  |  |  |
|  | Ma maladie serait difficile à supporter |  |  |  |  |  |
|  | Ma maladie aurait des conséquences importantes sur ma vie |  |  |  |  |  |
|  | Ma maladie n’aurait pas beaucoup d’effets sur ma vie |  |  |  |  |  |
|  | Ma maladie affecterait beaucoup la façon dont les autres me voient |  |  |  |  |  |
|  | Ma maladie entrainerait des problèmes financiers graves |  |  |  |  |  |
|  | Ma maladie causera des difficultés à mes proches |  |  |  |  |  |
|  | Il y a beaucoup de choses que je peux faire pour contrôler les symptômes |  |  |  |  |  |
|  | Ce que je fais peut déterminer l’amélioration ou l’aggravation de ma maladie |  |  |  |  |  |
|  | Le déroulement de ma maladie dépend de moi |  |  |  |  |  |
|  | Rien de ce que je fais n’affectera ma maladie |  |  |  |  |  |
|  | J’ai le pouvoir d’influencer ma maladie |  |  |  |  |  |
|  | Mes actions n’auront aucun effet sur l’évolution de ma maladie |  |  |  |  |  |
|  | Ma maladie va s’améliorer avec le temps |  |  |  |  |  |
|  | Il y a peu de choses à faire pour améliorer ma maladie |  |  |  |  |  |
|  | VOTRE POINT DE VUE  SUR VOTRE ’ULCERE DE BURULI | PAS DU TOUT D’ACCORD | PAS D’ACCORD | NI D’ACCORD NI PAS D’ACCORD | D’ACCORD | TOUT A FAIT D’ACCORD |
|  | ***Si j'aurais L'ulcère de Buruli ... ....*** | | | | | |
|  | Mon traitement sera efficace pour guérir ma maladie |  |  |  |  |  |
|  | Les effets négatifs de ma maladie peuvent être prévenus (évités) par mon traitement |  |  |  |  |  |
|  | Le traitement peut contrôler ma maladie |  |  |  |  |  |
|  | Malgré tout ce que je ferai mon état ne poura s’améliorer. |  |  |  |  |  |

|  | VOTRE POINT DE VUE  SUR VOTRE ’ULCERE DE BURULI | PAS DU TOUT D’ACCORD | PAS D’ACCORD | NI D’ACCORD NI PAS D’ACCORD | D’ACCORD | TOUT A FAIT D’ACCORD | |
| --- | --- | --- | --- | --- | --- | --- | --- |
|  | ***Si j'aurais L'ulcère de Buruli ... ....*** |  | | |  | | |
|  | Les symptômes de mon état me laissent perplexe |  |  |  |  |  | |
|  | Ma maladie est un mystère pour moi |  |  |  |  |  | |
|  | Je ne comprends pas ma maladie |  |  |  |  |  | |
|  | Ma maladie n’a aucun sens pour moi |  |  |  |  |  | |
|  | J’ai une image nette ou une compréhension de mon état |  |  |  |  |  |  |
|  | Les symptômes de ma maladie changent beaucoup d’un jour à l’autre |  |  |  |  |  |  |
|  | Les symptômes vont et viennent par cycles |  |  |  |  |  |  |
|  | ***Si j'aurais L'ulcère de Buruli ... ....*** | | | | | |  |
|  | Ma maladie est très imprévisible |  |  |  |  |  |  |
|  | Je passe par des cycles au cours desquels la maladie diminue ou empire |  |  |  |  |  |  |
|  | Je déprime quand je pense à ma maladie |  |  |  |  |  |  |
|  | Quant j’y pense, ma maladie m’inquiète |  |  |  |  |  |  |
|  | Ma maladie me met en colère |  |  |  |  |  |  |
|  | Ma maladie ne me gêne pas |  |  |  |  |  |  |
|  | A cause de cette maladie je suis anxieux |  |  |  |  |  |  |
|  | Ma maladie me fait peur |  |  |  |  |  |  |
|  | ***Si j'aurais L'ulcère de Buruli ... ....*** | | | | | |  |
|  | Traitement à un guérisseur traditionnel serait efficace pour guérir ma maladie. |  |  |  |  |  |  |
|  | Traitement dans un centre de soins de santé serait efficace pour guérir ma maladie. |  |  |  |  |  |  |
|  | Automédication serait efficace pour guérir ma maladie. |  |  |  |  |  |  |
|  | Les effets négatifs pourraient être évités par un traitement à un guérisseur traditionnel. |  |  |  |  |  |  |
|  | Les effets négatifs pourraient être évités par un traitement dans un centre de soins de santé. |  |  |  |  |  |  |
|  | Les effets négatifs pourraient être évités par l'automédication |  |  |  |  |  |  |

| LES CAUSES DE MA MALADIE  Imaginez que vous avez L'ulcère de Buruli. Nous sommes intéressés par ce que vous considérez la cause probable de votre ulcere de Buruli. Les gens étant différents, il n’y a pas de réponse correcte à cette question. Ce qui nous intéresse le plus c’est votre propre point de vue sur les facteurs qui ont causé votre ulcère de Buruli plutôt que ce que les autres, y compris les médecins ou la famille peuvent vous avoir suggéré. Ci-dessous figure une liste des causes possibles de votre ulcere de Buruli. |
| --- |

| CAUSES POSSIBLES | | PAS DU TOUT D’ACCORD | PAS D’ACCORD | NI D’ACCORD NI PAS D’ACCORD | D’ACCORD | TOUT A FAIT D’ACCORD |
| --- | --- | --- | --- | --- | --- | --- |
| 1. | Stress ou ennuis |  |  |  |  |  |
| 2. | Héréditaire – courant dans ma famille |  |  |  |  |  |
| 3. | Un microbe ou un virus |  |  |  |  |  |
| 4. | Régime ou habitudes alimentaires |  |  |  |  |  |
| 5. | Hasard ou malchance |  |  |  |  |  |
| 6. | Mauvais soins médicaux dans mon passé |  |  |  |  |  |
| 7. | Pollution de l’environnement |  |  |  |  |  |
| 8. | Mon propre comportement |  |  |  |  |  |
| 9. | Mon attitude mentale, ex. avoir une vision négative de la vie |  |  |  |  |  |
| 10. | Problèmes de famille ou soucis causés par ma maladie |  |  |  |  |  |
| 11. | Ma surcharge de travail |  |  |  |  |  |
| 12. | Mon état émotionnel, ex. abattement, solitude, anxiété, vide |  |  |  |  |  |
| 13. | Vieillissement |  |  |  |  |  |
| 14. | Consommation d’Alcool |  |  |  |  |  |
| 15. | Consommation de Tabac |  |  |  |  |  |
| 16. | Accident ou blessure |  |  |  |  |  |
| 17. | Ma personnalité |  |  |  |  |  |
| 18. | Détérioration de l’immunité |  |  |  |  |  |
| 19. | L'eau contaminée |  |  |  |  |  |
| 20. | Marche dans la boue |  |  |  |  |  |
| 21. | Piqûre d'un insecte |  |  |  |  |  |
| 22. | Vers aquatiques |  |  |  |  |  |
| 23. | Le manque d'hygiène |  |  |  |  |  |
| 24. | Sorcellerie |  |  |  |  |  |
| 25. | Les mauvais esprits |  |  |  |  |  |
| 26. | De Dieu |  |  |  |  |  |

Dans le tableau ci-dessous, veuillez classer dans l’ordre les trois facteurs les plus importants qui d’après vous ont causé votre ulcere de Buruli. Vous pouvez utiliser n’importe le quel des éléments de la case ci-dessus ou vous pouvez ajouter d’autres idées personnelles. ***Si j'aurais L'ulcere de Buruli, les causes les plus importantes pour moi seraient:***

1. _____________________________

2. _____________________________

3. _____________________________
